# Picocyanobacteria Community and Cyanophage Infection Responses to Nutrient Enrichment in a Mesocosms Experiment in Oligotrophic Waters

**DOI:** 10.3389/fmicb.2020.01153

**Published:** 2020-06-03

**Authors:** Alexandra Coello-Camba, Ruben Diaz-Rua, Carlos M. Duarte, Xabier Irigoien, John K. Pearman, Intikhab S. Alam, Susana Agusti

**Affiliations:** ^1^Red Sea Research Center, King Abdullah University of Science and Technology, Thuwal, Saudi Arabia; ^2^AZTI – Marine Research, Pasaia, Spain; ^3^IKERBASQUE, Basque Foundation for Science, Bilbao, Spain; ^4^Cawthron Institute, Nelson, New Zealand; ^5^Computational Bioscience Research Center, King Abdullah University of Science and Technology, Thuwal, Saudi Arabia

**Keywords:** *Synechococcus*, bloom, metagenomics, clade, cyanophages

## Abstract

*Prochlorococcus* and *Synechococcus* are pico-sized cyanobacteria that play a fundamental role in oceanic primary production, being particularly important in warm, nutrient-poor waters. Their potential response to nutrient enrichment is expected to be contrasting and to differ from larger phytoplankton species. Here, we used a metagenomic approach to characterize the responses to nutrient enrichment in the community of picocyanobacteria and to analyze the cyanophage response during a mesocosms experiment in the oligotrophic Red Sea. Natural picoplankton community was dominated by *Synechococcus* clade II, with marginal presence of *Prochlorococcus* (0.3% bacterial reads). Increased nutrient input triggered a fast *Synechococcus* bloom, with clade II being the dominant, with no response of *Prochlorococcus* growth. The largest bloom developed in the mesocosms receiving a single initial input of nutrients, instead of daily additions. The relative abundances of cyanophage sequences in cellular metagenomes increased during the experiment from 12.6% of total virus reads up to 40% in the treatment with the largest *Synechococcus* bloom. The subsequent collapse of the bloom pointed to a cyanophage infection on *Synechococcus* that reduced its competitive capacity, and was then followed by a diatom bloom. The cyanophage attack appears to have preferentially affected the most abundant *Synechococcus* clade II, increasing the evenness within the host population. Our results highlight the relevance of host-phage interactions on determining population dynamics and diversity of *Synechococcus* populations.

## Introduction

The cyanobacteria *Synechococcus* and *Prochlorococcus* are major components of marine pico-phytoplankton (<2 μm cell diameter). They play a key role in primary production in the oceans, particularly in warm and nutrient-poor waters (Partensky et al., 1999; Agawin et al., 2002), as their small size gives them a high specific affinity for the scarce nutrients (Raven, 1986; Chisholm, 1992; Raven, 1998). This high affinity and the fast growth of *Synechococcus*, also associated with its small size, allow these microorganisms to respond rapidly to nutrient inputs (Glover et al., 1988; Phlips et al., 1999; Agawin et al., 2000), thereby rendering it a major component of spring blooms in oligotrophic areas (Lindell and Post, 1995; DuRand et al., 2001). In contrast, increased nutrient concentrations have less conspicuous effects on the growth of its sister clade *Prochlorococcus* (Chung et al., 2011), that blooms in summer under very specific conditions of water stratification and nutrient depletion (Lindell and Post, 1995).

Genomic analyses have helped to identify at least 16 different *Synechococcus* clades (I–XVI) (Paerl et al., 2008; Ahlgren and Rocap, 2012; Huang et al., 2012; Mazard et al., 2012). There is a distinct distribution of *Synechococcus* clades globally, where clades I and IV usually dominate in cold, nutrient-rich waters; clades II, III, and X proliferate in warm and oligotrophic habitats; clades CRD1 and CRD2 are more successful in Fe-depleted waters, and the less abundant clades XV and XVI are mostly present at ecotone sites with intermediate conditions (Sohm et al., 2016). However, the genotypic composition of natural populations is dynamic and can change seasonally (Mackey et al., 2017; Ahlgren et al., 2019), as stratification and available nutrients vary along the year (Tai and Palenik, 2009; Post et al., 2011), or even at a shorter scale after temporary nutrient enrichments (Chung et al., 2011).

Due to the considerable biogeochemical and ecological significance of blooms, representing large biomass accumulations with impacts on carbon, nutrients and oxygen cycling, these phenomena have been an important subject of study since first described in the XIX Century (Francis, 1878). During the last years, innovative molecular tools, such as massively parallel DNA sequencing, have been used to allow a deeper understanding of the responses of marine microbial communities to different environmental conditions, unveiling the mechanisms responsible for population fluctuations (e.g., Chung et al., 2011; Post et al., 2011; Choi et al., 2013; Pearman et al., 2016).

*Synechococcus* blooms seasonally in the euphotic zone, when seawater temperature increases beyond a threshold value, and the nutrient availability is enhanced (Lindell and Post, 1995; Tai and Palenik, 2009; Chung et al., 2011; Post et al., 2011; Hunter-Cevera et al., 2016). Nutrient-induced *Synechococcus* blooming processes have been previously studied on natural samples during cruises, time series, and mesocosm experiments (Agawin et al., 2000; Tai and Palenik, 2009; Chung et al., 2011; Post et al., 2011), showing that *Synechococcus* can thrive shortly after nutrient addition, depending on nutrient loading (Agawin et al., 2000, 2004). Available results from mesocosm experiments showed the effect of nutrient addition on phytoplankton communities, mostly at a size-group or genus level. In the oligotrophic Mediterranean Sea, nutrient addition led to a fast response of picophytoplankton that was afterward replaced by microphytoplankton as the dominant group (Duarte et al., 2000). Agawin et al. (2004) also tested the effect of N:P ratios on Mediterranean picoplankton using nutrient enriched mesocosms. They observed that *Synechococcus* sp. and small flagellates dominated the water column 3 days after nutrient addition in all mesocosms, reaching different abundances depending on the N:P load. In fact, *Synechococcus* has a greater potential for nutrient acquisition in low-nutrient environments than bigger-celled photosynthetic plankton due to their small size and great ability to use different nitrogen sources (Raven, 1998; Moore et al., 2002). Amongst the different clades that constitute *Synechococcus* populations, those that consistently dominate the population throughout the year have also been shown to be dominant during the seasonal blooms (Tai and Palenik, 2009; Post et al., 2011), suggesting that these blooming processes do not preferentially affect particular clades.

Nutrient-induced increases in *Synechococcus* cell abundances trigger density-dependent mechanisms of population control (e.g., parasites and grazers) (Raven, 1998). These mechanisms decimate the blooming population, leading to a shift in the phytoplankton community composition toward the dominance of bigger cells as *Synechococcus* populations decline (Hein and Riemann, 1995; Duarte et al., 2000). Amongst these control mechanisms, viral infection has been defined as a key factor in determining host abundances and causing fast collapses of cyanobacteria blooms (Wilson and Mann, 1997; McDaniel et al., 2002; Mühling et al., 2005).

Viruses, mainly bacteriophages, are the most abundant entities in aquatic systems, reaching concentrations between 10^4^ and 10^8^ viruses mL^–1^ (Wommack and Colwell, 2000). Phages infecting cyanobacteria belong to the order Caudovirales, dsDNA tailed phages that include the families Myoviridae, Podoviridae and Siphoviridae (Mann, 2003; Ma et al., 2014). Cyanophages can have different host ranges, some of them showing preferences for specific host strains (e.g., Waterbury and Valois, 1993), therefore revealing a close relationship between the genetic diversities of both phage and host populations (Sullivan et al., 2003; Mühling et al., 2005; Gregory et al., 2016). Cyanophages are an abundant and dynamic component of marine waters (Bergh et al., 1989; Suttle and Chan, 1993, 1994), and when the host abundance surpasses a threshold value, viral propagation becomes more efficient and their concentration increases dramatically (Suttle and Chan, 1994; Weinbauer and Suttle, 1996).

Nutrient addition to experimental mesocosms can trigger phytoplankton blooms, followed by increased viral numbers and eventually a collapse of the bloom, as observed in *Emiliania huxleyi* (Bratbak et al., 1993; Jacquet et al., 2002) and *Synechococcus* (Wilson et al., 1998). However, McDaniel and Paul (2005) also tested the effect of nutrient addition in natural *Synechococcus* communities, finding a decrease in the lytic production. In mesocosms experiments with nutrient limitation, the growth of the host community was reduced, and viral production was delayed or reduced (Bratbak et al., 1993; Jacquet et al., 2002). The adsorption of cyanophages to host cells was observed to be independent of nutrient concentration in the medium (Wilson et al., 1996), suggesting that changes in host growth and abundance in response to nutrients increase will enhance cyanophage production (Middelboe, 2000; Mei and Danovaro, 2004).

The goal of this study was to characterize the response of a natural oligotrophic community of picocyanobacteria and its viruses to different nutrient loadings, reaching a deep taxonomic level through the use of metagenomic tools. We used mesocosms enclosures that allow reproducing the natural oligotrophic Red Sea environmental conditions and the responses and interactions of the whole plankton community. We expected that nutrient additions would stimulate cyanobacterial growth, generating changes in the proportions of *Synechococcus* and *Prochlorococcus* and in their population diversities, that would be unveiled through a metagenomic approach to a clade or subclade level. We also expected that the induction of host growth would stimulate lytic viral infection, leading to the collapse of the bloom. The analysis of metagenome would also characterize and reveal the changes experienced in viral taxonomic composition. This study, conducted through experimental manipulation of nutrients in mesocosms in the Red Sea, will help to understand picophytoplankton responses in a salty, warming, oligotrophic, and highly transparent ecosystem.

## Materials and Methods

### Experimental Set Up

The experimental setup and the evolution of the temperature, nutrients and overall conditions are described in detail in Pearman et al. (2016). Briefly, a set of mesocosm bags of 8000 L (2.5 m deep) was placed in the research harbor of King Abdullah University of Science and Technology (KAUST) in Thuwal, Saudi Arabia (22.3°N, 39.1°E), between the 27th January and the 15th February 2013. The experiment aimed to analyze the phytoplankton response to different nutrient loadings, as a single nutrient pulse (i.e., simulating a single event as a dust storm) and a continuous nutrient input (i.e., simulating a eutrophication source as coastal urbanization or aquaculture development) (Pearman et al., 2016). The experiment consisted of 4 nutrient treatments plus a control (no nutrient addition), with 2 replicates each (Table 1). In two treatments, a high nutrient pulse was added only once at the beginning of the experiment (initial addition treatments, NP-I and NPSi-I), while in the other two treatments, a lower concentration of nutrients was added every day over the first 2 weeks (continuous addition treatments, NP-C and NPSi-C) (Table 1).

**TABLE 1:** Characterization of the control and nutrient treatments tested in the mesocosms (NP-I, initial nitrate and phosphate addition; NPSi-I, initial nitrate, phosphate and silicate addition; NP-C, continuous initial nitrate and phosphate addition; NPSi-C, continuous nitrate, phosphate and silicate addition).

			**Nutrient treatment**			
		**Control**	**NP-I**	**NPSi-I**	**NP-C**	**NPSi-C**
Timing of nutrient addition		–	Single, at day 0	Single, at day 0	Daily, for 2 weeks	Daily, for 2 weeks
Concentration of nutrients added	NaNO_3_ (μM)	–	16	16	2	2
	H_2_NaO_4_P⋅H_2_O (μM)	–	1	1	0.12	0.12
	Na_2_SiO_3_⋅9H_2_O (μM)	–	–	39	–	3.75
Nutrients on day 1	NO_2_ + NO_3_ (μM)	0.1 (± 0.14)	11.19 (± 4.57)	10.54 (± 4.16)	3.09 (± 1.05)	3.81 (± 1.35)
	PO_4_ (μM)	✢	0.83 (± 0.39)	0.48 (± 0.18)	0.1 (± 0.14)	0.05 (± 0.07)
Nutrients on day 5	NO_2_ + NO_3_ (μM)	0.18 (± 0.03)	5 (± 1.48)	7.48 (± 2.19)	13.78 (± 4.06)	12.88 (± 2.72)
	PO_4_ (μM)	✢	0.34 (± 0.24)	0.53 (± 0.18)	0.93 (± 0.26)	0.73 (± 0.25)
Nutrient trends	NO_2_ + NO_3_ (d^–1^)	0.00 (± 0.01)^*ns*^	−1.02 (± 0.47)*	−1.31 (± 0.27)**	1.84 (± 0.48)*	−0.42 (± 1.02)^*ns*^
	PO_4_ (d^–1^)	✢	−0.06 (± 0.03)*	−0.03 (± 0.06)^*ns*^	0.12 (± 0.04)*	0.00 (± 0.07)^*ns*^
Maximum chlorophyll *a*	(μg L^–1^)	0.06 (± 0.01)	0.24 (± 0.1)	0.51 (± 0.22)	0.66 (± 0.59)	0.73 (± 0.8)

*The timing of nutrient inputs and concentrations added are indicated for each treatment. The nutrient concentrations measured for each treatment (average of replicated mesocosm bags with standard deviations) on days 1 and 5, and the mean trends in nutrient concentrations with time estimated during the first 2 weeks (± SD) are also indicated. Asterisks indicate p < 0.05; ns: not significant; ✢: below detection limits (0.01 μM). The maximum chlorophyll a concentrations measured during each incubation are also included (± SD).*

Temperature, salinity, and fluorescence profiles were measured daily using a CTD (Valeport Monitor CTD Profiler) with an attached chlorophyll sensor. Nitrate, nitrite, and orthophosphate concentrations were measured from each mesocosm bag, filtering through a 0.45 lm filter into acid washed sample bottles, and keeping samples stored at −20°C until analysis on an Autoanalyser AAIII pentacanal BRAN-LUEBBE (see Pearman et al., 2016 for details).

### Sampling

Surface water (1 m) was sampled daily from each mesocosm at solar noon, using a Niskin bottle. For *Synechococcus* cell counts, 15 mL of water pre-filtered through a 40 μm mesh were fixed in glutaraldehyde (2.5% final conc.) and flash frozen in liquid nitrogen until analysis. *Synechococcus* cell abundances were quantified using a FACSVerse flow cytometer (Becton Dickinson, Belgium), equipped with a blue laser (488 nm), using 1.002 μm beads (Polysciences, Europe) for verification of the equipment.

The net growth rates (μ) of *Synechococcus* along the experiment were calculated from the slope of changes of the natural logarithm of the cell abundance (N) over time (t, in days). To do so, we fitted the linear regression equation, where a equals the intercept of the line with cell abundance (y-axis):

$$Ln(N_{t})\;=\;a\;+\;\mu t$$

Water samples for metagenomic analysis were collected at the first day of incubation and at 2–3 day intervals, and immediately transferred to carboys for immediate filtration. Approximately 4 L of seawater was filtered through a 0.2 μm Cell-Trap^TM^ (Mem-Teq, United Kingdom). Concentrated cells were then eluted using 2 mL of filtered seawater (from the same sample), and immediately frozen in liquid nitrogen and stored at −80°C for later analysis. Results on total phytoplankton and plankton community responses are described in Pearman et al. (2016).

### DNA Extraction and Sequencing

Cells were pelleted via centrifugation and resuspended in buffer (Qiagen) and lysozyme. Cells were lysed using a Tissue Lyser II machine (Qiagen) and Zirconia/Silica Beads. DNA was extracted following a phenol: chloroform: isoamyl alcohol (IAA) method (see Pearman et al., 2016, for details).

Paired-end sequencing libraries (100 × 2 bp) were prepared following the manufacturer’s protocols using the NEBNext Ultra DNA kit (#E7370L). Six samples were multiplexed per lane and were subsequently sequenced on an Illumina HiSeq 2000 sequencer at the KAUST Biosciences Corelab (BCL).

### Sequence Analysis

The analysis of the metagenomic data obtained here was performed in the following manner. High quality reads and corresponding assemblies were obtained from every sample using standard error-correction methods, followed by assembly procedure using metaSpades Software v3.9.0 (Nurk et al., 2017), and resulting contigs were filtered for minimum length of 500. Gene prediction was performed using Prodigal with options “-p meta” and the “-c” switch for closed ends. Clustering of gene coding sequences (CDSes) by pooling all genes from all samples was performed using CD-HIT producing a non-redundant gene catalog. Paired-end reads from individual samples were mapped on to the gene catalog to seek gene presence and abundance estimates. Read mapping was performed using standard bow-tie software parameters by providing error-corrected paired end reads and resulting alignments in sam format were processed through eXpress software to obtain normalized mapped reads as reads (transcripts) per million and Fragment Per Kilobase per Million (FPKM) reads.

For annotation and visualization, we used Annotation and Compare modules provided by the Dragon Metagenomic Analysis Platform (DMAP)^1^. The Annotation module allowed a comprehensive annotation of genes based on reference datasets employing Automatic Annotation of Microbial or Meta Genomes (AAMG). AAMG provided taxonomic assignments based on traversing BLAST results for Lowest Common Ancestor (LCA) approach (considering a minimum *E*-value of 1E-2 and 50% of BLAST coverage).

The Compare module provided an interface to compare and visualize samples based on available taxonomic and functional annotation.

We filtered data for *Synechococcus* (taxon id 1129) in DMAP using filter blast_tree:1129. The normalized read counts for genes related to different *Synechococcus* strains were manually assigned to their correspondent clade in agreement with the databases compiled in previous works (Mazard et al., 2012; Farrant et al., 2016). Also using DMAP, we looked for the single-copy, ribosomal protein gene *rpsC* amongst *Synechococcus* data (blast_tree:1129, HMM accession id: PF000189 and 80% blast coverage), to relate the normalized read counts of this target gene with the *Synechococcus* cell abundances determined with flow cytometry.

To look for related phage presence, absence or abundance in our metagenomic samples, we followed the same process as for *Synechococcus*, using the corresponding taxon id for all viruses (10239), and for the families Myoviridae (10662), Siphoviridae (10699), and Podoviridae (10744), and selecting those phages classified as *Cyanobacteria*, *Prochlorococcus* or *Synechococcus* phages.

To determine population evenness (Shannon’s equitability, E_*H*_), we estimated the Shannon diversity index (H) within the *Synechococcus* populations from the different treatments using the alpha_diversity.py script in QIIME, and then applied the formula:

(1) E=HH/Hm⁢a⁢x

where H_*max*_ is the logarithm of the number of *Synechococcus* clades (clade richness, S) found in each sample.

## Results

Nutrient concentrations were low at the onset of the experiment, with nitrite plus nitrate concentrations of 0.1 μM and phosphate below detection levels (detection limit for phosphate: 0.01 μM, Table 1). The initial addition in the NP-I and NPSi-I treatments led to a strong increase in nutrient concentrations on day 1, decreasing over time (Table 1). Nutrient concentrations in the NP-C and NPSi-C treatments were low on day 1, but accumulated in the mesocosms during the 2 weeks of continuous addition (Table 1). Throughout the incubation time, average (± SE) values for temperature and salinity in all mesocosms were 25 (± 0.3) °C and 40.4 (± 0.1) psu, respectively.

Chlorophyll *a* concentrations remained low in the control treatment (Table 1), and showed the highest values in the continuous addition treatments on days 13 (NP-C) and 8 (NPSi-C). In the NP-I and NPSi-I treatments the maximum chlorophyll *a* concentrations were reached on days 5 and 8, respectively (Table 1).

**FIGURE 1:**
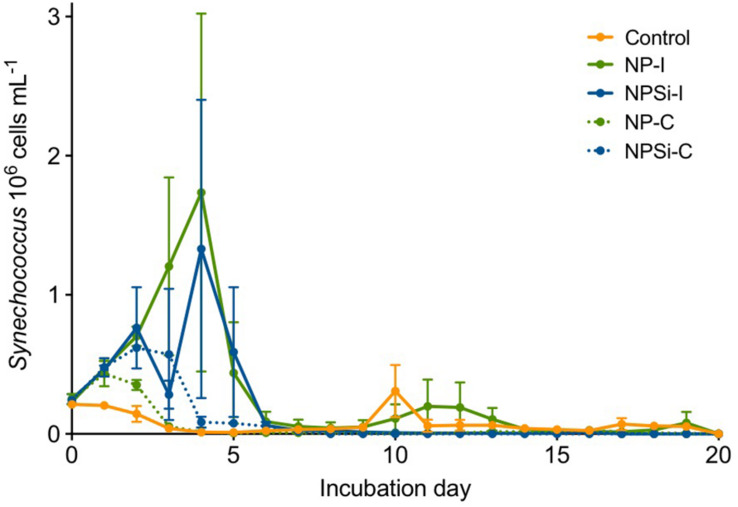
*Synechococcus* cell abundances (in 10^6^ cells mL^–1^, ± range) determined along the duration of the experiment for control and nutrient addition treatments (NP-I, initial nitrate and phosphate addition; NPSi-I, initial nitrate, phosphate and silicate addition; NP-C, continuous initial nitrate and phosphate addition; NPSi-C, continuous nitrate, phosphate and silicate addition).

*Synechococcus* dominated the initial pico-phytoplankton community, with an abundance of 0.24 × 10^6^ cells mL^–1^ (Figure 1). *Prochlorococcus* was not detected in the flow cytometric counts for the initial community or along the experiment. During the first day of incubation, the growth rate (± SE) estimated for the control treatment was -0.02 d^–1^ (± 0.05, *p* = 0.76), as *Synechococcus* cell abundance decreased in the absence of nutrient addition (Figure 1).

The growth during the first day of incubation was similar between the four nutrient addition treatments, averaging 0.28 d^–1^ (± 0.03, *p* < 0.0001). In the treatments receiving continuous nutrient input, *Synechococcus* abundances peaked between days 1 and 2, reaching maximum values of 0.43 × 10^6^ cells mL^–1^, and 0.62 × 10^6^ cells mL^–1^ in the NP-C and NPSi-C treatments, respectively (Figure 1). However, in the treatments receiving a single initial nutrient pulse, *Synechococcus* reached the maximum cell concentrations at day 4, of 1.73 × 10^6^ cells mL^–1^ for NP-I, and 1.33 × 10^6^ cells mL^–1^ for NPSi-I treatments (Figure 1). After reaching the highest abundances, *Synechococcus* populations decreased in all the mesocosms and remained at low concentrations after day 6, with only a small increase during the second week in the control and NP-I treatment (Figure 1).

### *Synechococcus* Genes and Clade Composition

Total hit numbers assigned to different taxonomic levels are shown in Table 2. At the beginning of the experiment, the proportion of bacterial reads assigned to *Synechococcus* averaged 15.7%. Its sister genus *Prochlorococcus* was also detected, but only averaged 0.3% of bacterial reads (Table 2). Within the total virus community, a high proportion of the reads was assigned to the Myoviridae family (average 34.6%). For the Podoviridae and Siphoviridae families, these proportions were lower (3.9 and 5.2%, respectively).

**TABLE 2:** Gene distribution based on taxonomic assignment, indicated as number of hits (in bold) for bacteria, archaea, eukaryotes and viruses, and as percentage of total bacterial or viral reads for *Prochlorococcus, Synechococcus*, Myoviruses, Podoviruses and Siphoviruses (indicated as mean values ± standard errors).

**Treatment**	**Day**	**Bacteria**	**(± SE)**	**% *Pro.***	**(± SE)**	**% *Syn.***	**(± SE)**	**Archaea**	**(± SE)**	**Eukaryota**	**(± SE)**	**Viruses**	**(± SE)**	**% Myov.**	**(± SE)**	**% Podov.**	**(± SE)**	**% Siphov.**	**(± SE)**
**Control**	1	**554840**	**49153**	0.29	0.03	16.37	1.17	**926**	**109**	**19857**	**592**	**12144**	**2785**	36.73	0.71	4.04	0.02	4.26	0.32
	5	**674369**	**–**	0.05	**–**	2.91	**–**	**872**	**–**	**25800**	**–**	**7063**	**–**	38.57	**–**	5	**–**	5.18	**–**
	6	**687281**	**363**	0.09	0.02	6.91	3.44	**782**	**50**	**35167**	**1629**	**7162**	**585**	36.65	1.88	5.42	0.18	5.44	0.16
	8	**482587**	**28003**	0.11	0.01	9.04	1.6	**838**	**158**	**57617**	**931**	**17658**	**4625**	42.77	9.08	4.57	1.8	4.15	0.75
	10	**376295**	**–**	0.07	**–**	2.33	**–**	**945**	**–**	**58117**	**–**	**68633**	**–**	52.41	**–**	3.2	**–**	4.27	**–**
	13	**586516**	**34535**	0.23	0.13	14.47	7.17	**1068**	**312**	**57434**	**162**	**14662**	**9605**	27.18	5.8	6.17	0.44	4.58	0.58
	20	**503209**	**47585**	0.26	0.13	15.02	5.41	**923**	**91**	**52664**	**663**	**19814**	**3063**	23.82	0.53	8.64	0.71	4.95	0.69
**NP-I**	1	**691126**	**949**	0.24	0.06	14.7	1.47	**1195**	**155**	**13030**	**1823**	**11753**	**2421**	36.04	0.54	3.39	0.12	6.36	0.09
	5	**554802**	**18494**	0.4	0.02	25.92	1.2	**1283**	**92**	**21824**	**1503**	**122259**	**17223**	60.77	1.02	1.55	0.06	3.27	0.07
	6	**551872**	**36668**	0.23	0.07	18.35	6.98	**1794**	**566**	**18711**	**6419**	**148360**	**66868**	53.38	3.58	2.22	0.12	3.19	0.29
	8	**542814**	**51588**	0.12	0.02	5.47	2.87	**1907**	**510**	**15744**	**7719**	**140927**	**10244**	53.78	0.04	2.4	0.44	3.49	0.2
	10	**373536**	**–**	0.2	**–**	16.87	**–**	**1820**	**–**	**39085**	**–**	**137858**	**–**	45.89	**–**	3.04	**–**	5.77	**–**
	13	**575368**	**27995**	0.19	0.15	13.13	12.02	**1439**	**452**	**45960**	**24090**	**75086**	**30775**	36.17	3.24	6.17	2.67	4.47	0.82
	16	**555404**	**2808**	0.1	0.02	6.67	2.85	**3759**	**2335**	**25963**	**4335**	**86638**	**28579**	42.83	4.09	4.78	0.81	4.81	0.18
	20	**564113**	**50611**	0.11	0.07	6.24	5.6	**14893**	**12625**	**23126**	**3355**	**48584**	**6809**	37.61	8.13	8.34	2.79	5.18	0.38
**NPSi-I**	1	**527951**	**–**	0.29	**–**	14.97	**–**	**1854**	**–**	**40571**	**–**	**10271**	**–**	37.23	**–**	4.09	**–**	6.44	**–**
	5	**612034**	**16854**	0.41	0.12	27.1	2.64	**1616**	**307**	**22065**	**3664**	**89168**	**33892**	53.53	8.83	2.05	0.8	3.36	0.43
	6	**527686**	**–**	0.23	**–**	18.22	**–**	**1613**	**–**	**8194**	**–**	**219876**	**–**	59.07	**–**	1.55	**–**	2.24	**–**
	8	**349136**	**–**	0.2	**–**	5.76	**–**	**2423**	**–**	**25747**	**–**	**273292**	**–**	54.5	**–**	1.82	**–**	2.8	**–**
	10	**398572**	**–**	0.14	**–**	2.65	**–**	**2321**	**–**	**24274**	**–**	**250704**	**–**	51.28	**–**	1.91	**–**	3.24	**–**
	13	**500199**	**7574**	0.06	0.02	1.06	0.79	**1598**	**501**	**46199**	**20969**	**99505**	**56685**	47.04	0.69	4.35	1.33	4.5	0.28
	16	**688803**	**–**	0.04	**–**	0.17	**–**	**1451**	**–**	**11379**	**–**	**56000**	**–**	40.93	**–**	6.24	**–**	4.42	**–**
	20	**471425**	**30545**	0.05	0.01	0.27	0.07	**1809**	**886**	**48129**	**25323**	**81730**	**54917**	36.91	5.65	6.35	1.19	4.78	0.2
**NP-C**	1	**680526**	**52798**	0.32	0.01	16.16	0.2	**1227**	**86**	**18430**	**7105**	**16561**	**6484**	28.13	3.32	4.19	1.05	4.42	0.32
	5	**702845**	**–**	0.07	**–**	3.48	**–**	**13230**	**–**	**31724**	**–**	**33344**	**–**	32.64	**–**	6.23	**–**	5.63	**–**
	6	**578922**	**84942**	0.05	0.01	1.62	0.24	**1775**	**646**	**43237**	**18632**	**25368**	**2989**	31.37	1.73	6.97	0.91	4.95	0.27
	8	**575571**	**125550**	0.05	0	0.98	0.53	**1035**	**129**	**18158**	**9166**	**27027**	**4634**	25.38	2.38	10.75	1.44	6.05	0.18
	10	**368370**	**29105**	0.06	0.01	1.48	1.2	**1072**	**266**	**47381**	**11469**	**44932**	**18433**	26.52	10.46	6.76	1.5	5.29	0.75
	13	**495679**	**76171**	0.02	0.01	0.42	0.21	**1046**	**364**	**57748**	**28795**	**23227**	**2473**	14.51	3.48	12.86	3.7	4.69	1.73
	16	**573138**	**–**	0.03	**–**	1.96	**–**	**1399**	**–**	**34939**	**–**	**26055**	**—-**	13.76	**–**	11.58	**–**	3.66	**–**
	20	**389878**	**–**	0.02	**–**	0.23	**–**	**1081**	**–**	**59914**	**–**	**21138**	**–**	14.48	**–**	17.98	**–**	5.71	**–**
**NPSi–C**	1	**537071**	**50375**	0.32	0.11	16.3	1.18	**1231**	**402**	**23797**	**5206**	**16677**	**4102**	35.02	1.39	3.77	0.24	4.68	0.38
	5	**407588**	**132060**	0.15	0.04	10.09	1.62	**10135**	**8267**	**174427**	**58433**	**41400**	**2897**	47.72	0.64	3.31	0.13	5.1	0.18
	6	**372111**	**–**	0.09	**–**	6.5	**–**	**1849**	**–**	**148670**	**–**	**48670**	**–**	50.72	**–**	3.56	**–**	4.37	**–**
	8	**446293**	**63938**	0.05	0.01	0.98	0.34	**1091**	**133**	**124627**	**18898**	**46447**	**9257**	39.72	6.11	9.53	2.35	4.82	0.19
	10	**540147**	**134340**	0.04	0.02	0.09	0.04	**1171**	**2**	**68771**	**52692**	**37720**	**17053**	37.72	3.85	8.59	0.15	5.19	0.02
	13	**559486**	**36162**	0.02	0	0.06	0.03	**1238**	**98**	**37025**	**4749**	**24551**	**3107**	16.5	7.3	9.33	0.63	4.15	0.37
	16	**643410**	**–**	0.02	**–**	0.04	**–**	**2157**	**–**	**8772**	**–**	**40602**	**–**	10.01	**–**	8.24	**–**	2.98	**–**
	20	**598247**	**10645**	0.03	0	0.07	0.01	**1705**	**273**	**24133**	**14081**	**47445**	**21006**	24.07	0.42	13.03	0.37	4.87	0.27

**FIGURE 2:**
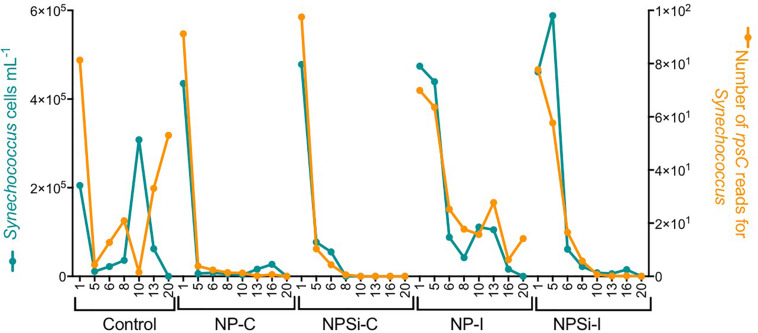
Relationship between the cell abundance measured using flow cytometry and the number of *rpsC* reads for *Synechococcus*, determined during our experiment and broken down for each treatment and day.

For *Synechococcus*, we observed a strong positive relationship between the number of *rpsC* reads taxonomically assigned to *Synechococcus* and the cell abundances determined with flow cytometry (*R*^2^ = 0.68, *p* < 0.0001, Figure 2).

**FIGURE 3:**
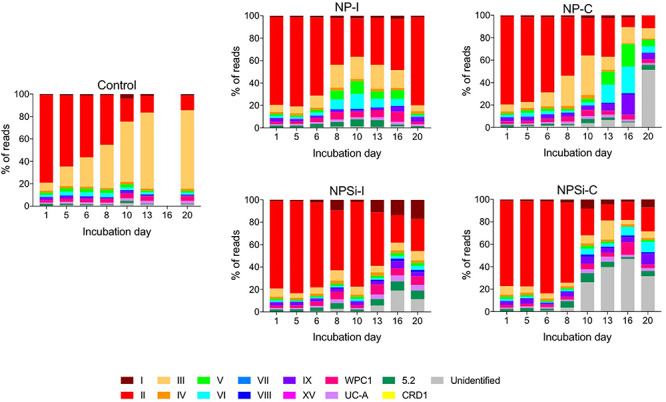
Changes over time in the different clades of *Synechococcus* found for control and nutrient addition treatments, according to the relative count of gene reads (%). Each color corresponds to a different clade; unidentified reads are represented as “unidentified” and colored gray (see legend).

Amongst the *Synechococcus* strains obtained by DMAP analysis, 43 were assigned to 14 different clades, based on Mazard et al. (2012) and Farrant et al. (2016) (Table 3). Other strains found could not be assigned to any specific clade, and were grouped together as “Unclassified” (Table 3). The highest proportion of reads corresponded to clades II and III. Gene reads related to clades I, IV, V, VI, VIII, IX, UC-A, and WPC1 were also present, with clades CRD1, VII and XV, and members of the 5.2 sub-cluster being the least abundant. At the beginning of the incubations, gene reads assigned to clade II were dominant in all treatments, comprising more than a 76% of the total *Synechococcus* reads (Figure 3). More specifically, strains WH 8109 (subclade IIb) and CC9605 (subclade IIc) stood out among the most abundant in all experiments. The second most represented clade (clade III), comprised between 6 and 8% of total *Synechococcus* reads at the first day of incubation in all treatments (Figure 3).

**TABLE 3:** Taxonomically assigned *Synechococcus* strains grouped by their correspondent clade, following the databases presented in Mazard et al. (2012) and Farrant et al. (2016).

**Clade**	**Strain**	**Clade**	**Strain**
5.2	GFB01	VIII	RS9917
	Minos11		WH 8101
	RCC307	IX	RS9916
	WH 5701	XV	UW69
CRD1	BIOS-E4-1	UC-A	KORDI-100
	BIOS-U3-1	WPC1	KORDI-49
I	CC9311		
	MVIR-16-2		
	MVIR-18-1	**“Unclassified” strains**	
	RCC328	ARC-11	
	WH 8016	*Candidatus* “Synechococcus spongiarum”	
	WH 8020	JA-2-3B’a(2-13)	
II	A15-44	JA-3-3Ab	
	A15-62	M11.2	
	CC9605	PCC 6312	
	KORDI-52	PCC 7002	
	M16B.1	PCC 7335	
	PROS-U-1	PCC 7502	
	RCC374	Syn01/0201	
	RS9907	Syn01/0202	
	TAK9802	*Synechococcus elongatus*	
	UW122	UH7	
	WH 8012	Uncultured marine type-A *Synechococcus* 4O4	
	WH 8109	Uncultured marine type-A *Synechococcus* 5B2	
III	A15-24.2	Uncultured marine type-A *Synechococcus* GOM 3M9	
	RS9915	Uncultured marine type-A *Synechococcus* GOM 3O12	
	WH 8102	Uncultured marine type-A *Synechococcus* GOM 3O6	
	WH 8103	Uncultured marine type-A *Synechococcus* GOM 4N23	
IV	BL107	Uncultured marine type-A *Synechococcus* GOM 4P21	
	CC9902	Uncultured marine type-A *Synechococcus* GOM 5D20	
V	BMK-MC-1	Uncultured *Synechococcus* sp.	
	WH 7803		
VI	MEDNS5		
	WH 7805		
VII	A15-60		
	NOUM97013		
	RCC67		

The changes in the proportion of reads assigned to the different *Synechococcus* clades was analyzed for the first 2 weeks of incubation (Table 4), as the decrease in total reads assigned to *Synechococcus* obtained for subsequent days was too low to reliably assign the sequences. The lower number of reads and their more even dispersion among multiple clades made difficult their identification, and was reflected here in an increase in the proportion of reads related to unidentified *Synechococcus* toward the end of incubation, significantly in treatments NP-C and NPSi-C treatments receiving continuous nutrient additions (Figure 3 and Table 4). The proportion of the most abundant clade (clade II) decreased with time along the first 2 weeks, significantly in all treatments except for NPSi-I (*t*-test, Figure 3 and Table 4). In the initial addition treatment but without Si (NP-I), we observed a significant increase in the proportion of clades III, WPC1, UC-A and sub-cluster 5.2. In both continuous addition treatments, both clade UC-A and the unidentified *Synechococcus* proportions increased significantly with time (*t*-test, Figure 3 and Table 4). Also, clades I and VIII increased significantly in NP-C mesocosms (*t*-test, Figure 3 and Table 4). In the control mesocosm clade IX also decreased, with a significant increase in the proportion of clades III, WPC1 and UC-A (*t*-test, Figure 3 and Table 4).

**TABLE 4:** Slopes of the change in the read proportions for the different *Synechococcus* clades with time in days (d^–1^) for each treatment, estimated during the first 2 weeks of incubation.

				**Initial nutrient addition**						**Continuous nutrient addition**					
	**Control**			**NP-I**			**NPSi-I**			**NP-C**			**NPSi-C**		
	**Mean**	**± S.E.**	***p***	**Mean**	**± S.E.**	***p***	**Mean**	**± S.E.**	***p***	**Mean**	**± S.E.**	***p***	**Mean**	**± S.E.**	**p**
I	0.12	0.12	*^*ns*^*	0.10	0.04	*^*ns*^*	0.77	0.39	*^*ns*^*	0.11	0.02	*	0.47	0.23	*^*ns*^*
II	–5.77	0.63	*	–4.17	1.33	*	–2.43	1.24	*^*ns*^*	–4.45	0.89	*	–5.99	2.00	*
III	5.39	0.49	*	1.54	0.40	*	0.02	0.22	*^*ns*^*	1.44	1.26	*^*ns*^*	0.66	0.48	*^*ns*^*
IV	0.02	0.03	*^*ns*^*	0.06	0.04	*^*ns*^*	0.08	0.05	*^*ns*^*	0.06	0.07	*^*ns*^*	0.00	0.03	*^*ns*^*
V	–0.04	0.08	*^*ns*^*	0.68	0.34	*^*ns*^*	0.08	0.04	*^*ns*^*	0.69	0.31	*^*ns*^*	0.03	0.06	*^*ns*^*
VI	–0.02	0.10	*^*ns*^*	0.84	0.39	*^*ns*^*	0.04	0.04	*^*ns*^*	0.95	0.48	*^*ns*^*	0.19	0.17	*^*ns*^*
VII	0.00	0.00	*^*ns*^*	0.00	0.00	*^*ns*^*	0.00	0.00	*^*ns*^*	0.00	0.00	*^*ns*^*	0.00	0.00	*^*ns*^*
VIII	0.03	0.01	*^*ns*^*	0.04	0.02	*^*ns*^*	0.06	0.02	*^*ns*^*	0.04	0.01	*	0.10	0.06	*^*ns*^*
IX	–0.14	0.05	*	–0.10	0.04	*^*ns*^*	0.04	0.03	*^*ns*^*	0.21	0.11	*^*ns*^*	0.18	0.16	*^*ns*^*
XV	0.00	0.00	*^*ns*^*	0.00	0.00	*^*ns*^*	0.00	0.00	*^*ns*^*	0.00	0.00	*^*ns*^*	0.00	0.00	*^*ns*^*
WPC1	0.18	0.03	*	0.39	0.13	*	0.50	0.25	*^*ns*^*	0.20	0.11	*^*ns*^*	0.17	0.10	*^*ns*^*
UC-A	0.14	0.03	*	0.12	0.03	*	0.20	0.10	*^*ns*^*	0.13	0.02	*	0.28	0.08	*
5.2	–0.06	0.07	*^*ns*^*	0.44	0.11	*	0.27	0.17	*^*ns*^*	0.11	0.10	*^*ns*^*	0.42	0.23	*^*ns*^*
CRD1	0.00	0.00	*^*ns*^*	0.00	0.00	*^*ns*^*	0.00	0.00	*^*ns*^*	0.00	0.00	*^*ns*^*	0.00	0.00	*^*ns*^*
Unidentified	0.14	0.07	*^*ns*^*	0.06	0.03	*^*ns*^*	0.38	0.17	*^*ns*^*	0.52	0.10	*	3.49	1.02	*

A t-test was applied to determine statistically significant results (ns: not significant; *p < 0.05).

**FIGURE 4:**
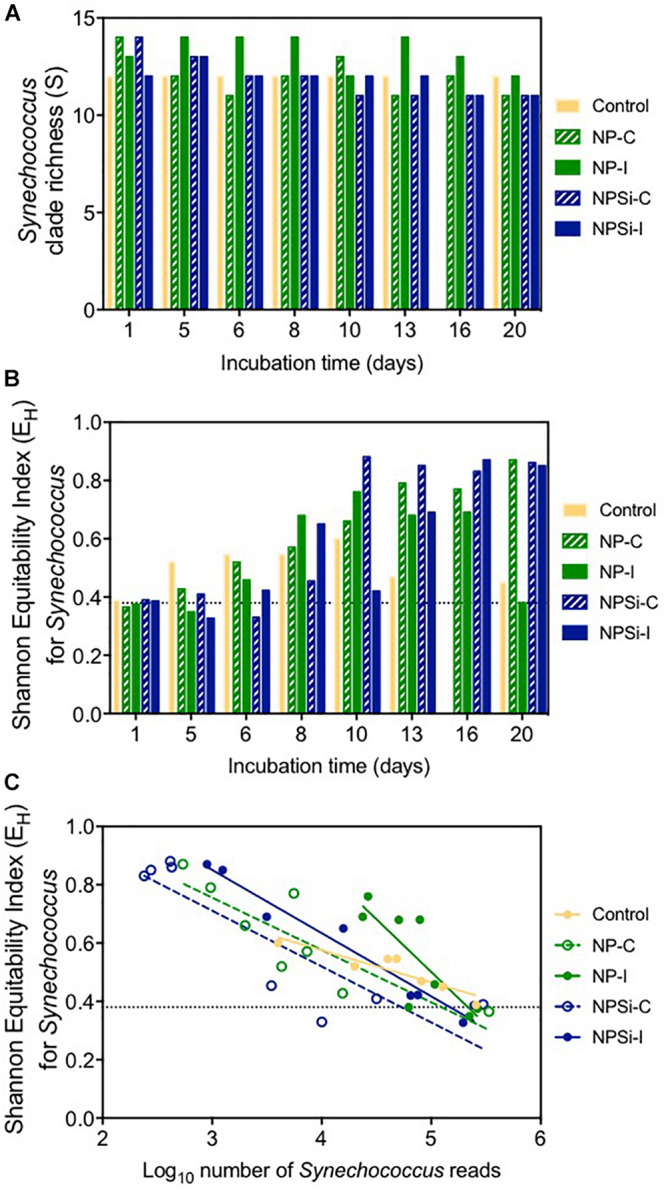
**(A)** Changes in *Synechococcus* clade richness with time (in days). Dashed line indicates the average richness at the beginning of the experiment. **(B)** Changes in Shannon’s equitability for *Synechococcus* with time (in days). Dashed line indicates the value for this index at the beginning of the experiment. **(C)** Relationship between Shannon’s equitability and the number of reads (in Log_10_) for *Synechococcus*.

After 1 day of incubation, the number of different clades identified in each treatment (clade richness) varied slightly between 12 clades in the control and NPSi-I treatments to 14 in the continuous nutrient addition treatments (Figure 4A). Clade richness was higher in the NP-I treatment during most of the experimental time, decreasing at the end of the experiment, as observed for all nutrient addition treatments (Figure 4A). The number of clades in the control treatment remained at 12 during all the experiment (Figure 4A).

The clade evenness within the *Synechococcus* populations represented using Shannon’s equitability showed in general the opposite tendency (Figure 4B). The clade evenness of the *Synechococcus* population increased over time, although for the control and NP-I treatments decreased toward the end of the experiment (Figure 4B), following the changes in the proportion of clade II along the experiment. For the rest of the treatments, E_*H*_ showed a tendency to increase with time, showing high values close to 0.8 at the end of the experiment (Figure 4B). When compared to the number of reads assigned to *Synechococcus*, Shannon’s equitability value increased in all treatments as the number of *Synechococcus* reads decreased (Figure 4C), suggesting that the increase in evenness is related to the decrease in *Synechococcus* populations.

### Responses of Cyanophages and Virus

**FIGURE 5:**
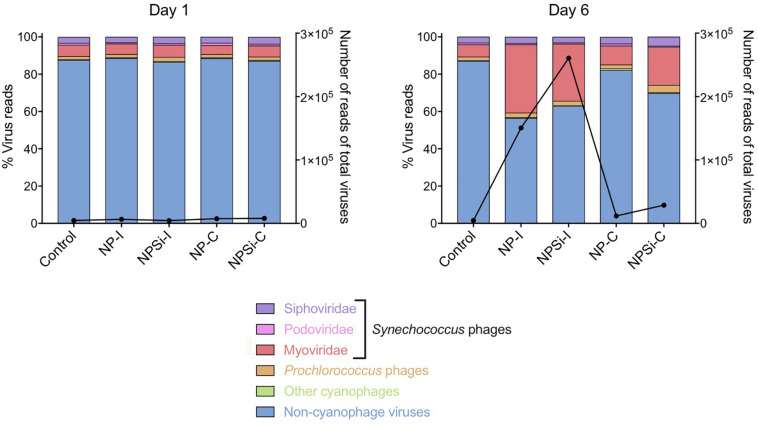
Changes in the relative proportions of reads assigned to *Synechococcus* phages and other viruses on days 1 and 6 under each treatment. Viral reads assigned to cyanophages, but with primary host unidentified to genus level are represented as “Other cyanophages.” The black line represents the number of reads of all virus present.

The cyanophage community represented a small proportion (12.6%) of the total number of viral reads at the beginning of the experiment (Figure 5). This proportion increased along the experiment, showing the maximum values on day 5 in the NP-C treatment (20.5%), and on day 6 in NPSi-C (30.3%), NP-I (43.6%), and NPSi-I (37.1%). In the control treatment, the proportion of cyanophages amongst the total viral reads also increased, but the maximum values were reached later in the experiment (day 10, 34%). We identified 53 different cyanophages after the taxonomic assignment of our metagenomic data, able to infect *Synechococcus*, *Prochlorococcus*, or both (Table 5). Amongst the *Synechococcus* phages, 14 were T4-like viruses from the Myoviridae family, 17 belong to the Podoviridae family, and 5 to Siphoviridae. The T4-like phages were the most abundant (e.g., *Synechococcus* phages S-RSM4 and ACG-2014h), in terms of number of reads, followed by members of the Siphoviridae family (e.g., *Synechococcus* phage S-SKS1). Podoviruses were also present, including several T7-like viruses (Table 5), but were much less represented.

**TABLE 5:** Cyanophages identified along the experiment, grouped by Family and preferred host.

**Family**	**Phage**	**Family**	**Phage**
**Host**: ***Synechococcus***		**Host**: ***Prochlorococcus***	
Myoviridae	ACG-2014b	Myoviridae	P-SSM2
	ACG-2014c		P-SSM4
	ACG-2014d	Podoviridae	P-SSP10
	ACG-2014e		P-SSP7
	ACG-2014f		T7-like virus 10G
	ACG-2014g		T7-like virus P-SSP2
	ACG-2014h		T7-like virus P-SSP6
	ACG-2014i		T7-like virus P-TIP44
	ACG-2014j	**Host**:***Synechococcus***	
	metaG-MbCM1	**and *Prochlorococcus***	
	S-MbCM100	Myoviridae	8B026
	S-PM2	Podoviridae	9515-10a
	S-RSM4		NATL1A-7
	syn9		NATL2A-133
Podoviridae	P60		PP
	S-CBP1		PSS2
	S-CBP4		S-CBP1
	S-CBP42		S-CBP2
	S-RIP1		S-CBP3
	S-RIP2		
	Syn5		
	T7-like virus 303		
	T7-like virus B4-1		
	T7-like virus B8-2		
	T7-like virus E3-1		
	T7-like virus F5-1		
	T7-like virus oc24e		
	T7-like virus oc43		
	T7-like virus S-TIP30		
	T7-like virus S-TIP37		
	T7-like virus S-TIP41		
Siphoviridae	S-CBS1		
	S-CBS2		
	S-CBS3		
	S-CBS4		
	S-SKS1		

**FIGURE 6:**
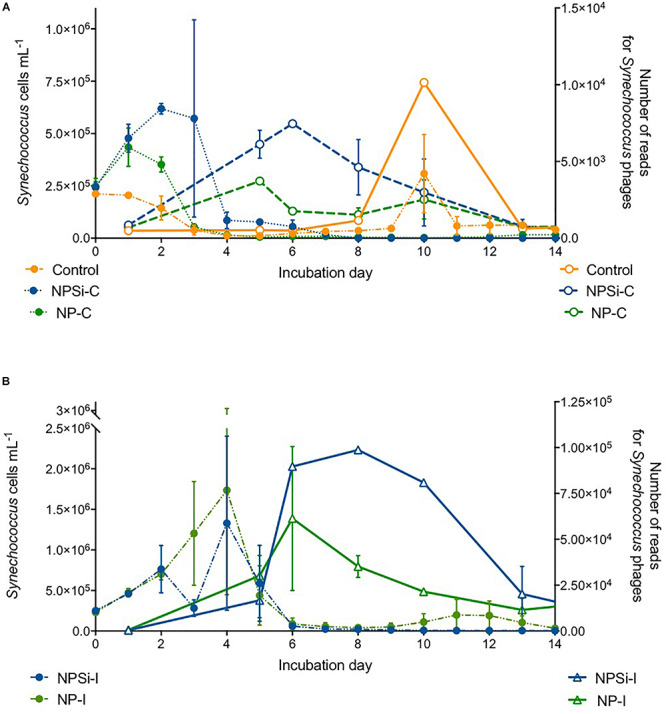
Comparison between the timing of *Synechococcus* cell abundances (cells mL^–1^) and number of *Synechococcus* phage reads along the experiment, estimated as mean values for the duplicate mesocosms (± standard errors), for the control and continuous treatments **(A)**, and for the initial treatments **(B)**.

The proportion of reads for *Synechococcus* phages increased a few days after the peak in *Synechococcus* cell abundances (Figures 6A,B). We observed a slight increase in the proportion number of reads for *Synechococcus* phages on the control and continuous treatments (Figure 6A), and a stronger increase in NP-I and NPSi-I, reaching the highest values at days 6 and 8, respectively (Figure 6B).

**FIGURE 7:**
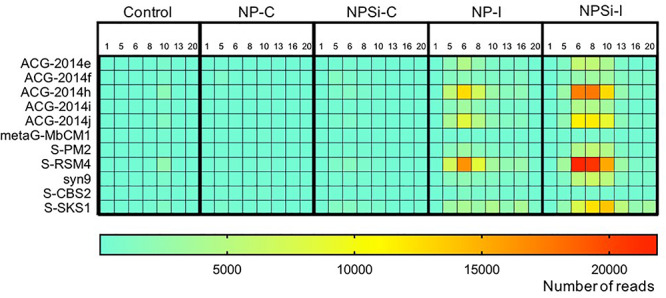
Heatmap showing the number of reads (average values from duplicate mesocosm bags) for the most abundant *Synechococcus* phages for all treatments, from days 1 to 20.

The *Synechococcus* phages that contributed most to this increase in read counts belonged to the Myoviridae family, with the proportion of reads assigned to this family increasing significantly from an average 5.8% (± SE = 0.3) on day 1 to 24.5% (± SE = 5.8) on day 6, for the nutrient addition treatments (Pearson’s Chi-squared test, *p* < 0.001, Figures 5, 7).

## Discussion

Our results, by using a metagenomic approach, showed the effects of nutrient enrichment on a *Synechococcus* community at a low taxonomic level and cyanophages, during a mesocosm experiment with warm and oligotrophic waters of the Red Sea.

Our data showed that *Synechococcus* populations dominated the phytoplankton response during the first days after nutrient addition. At the beginning of the experiment, *Synechococcus* cell abundances fell within the range of values previously reported for the Red Sea (Veldhuis and Kraay, 1993; Kürten et al., 2015). During the first days of incubation, *Synechococcus* responded rapidly to the nutrient input, consistent with the occurrence of *Synechococcus* blooms in areas subject to episodic nutrient pulses (Phlips et al., 1999). In fact, pico-sized autotrophic plankton can experience nutrient limitation in oligotrophic waters (Vaulot et al., 1996; Thingstad, 1998), where they can typically support relatively fast, even if suboptimal, growth rates due to their high specific affinity for nutrients (Chisholm, 1992; Vaulot et al., 1996; Raven, 1998). The nutrient-stimulated growth of *Synechococcus* was more intense in the treatments with a large initial nutrient pulse, indicating that the higher nutrient loading allowed a more sustained growth than in the continuous addition treatments, reaching higher cell abundances (Agawin et al., 2000). In addition, the rapid growth and nutrient uptake rates that characterize *Synechococcus*, provided an advantage in responding to a nutrient pulse, whereas larger phytoplankton, including diatoms, would be better able to compete when nutrients are supplied continuously (Pearman et al., 2016). This was supported by chlorophyll *a* concentrations, that peaked after the first week of incubation reflecting an increase in total autotrophic biomass, mainly diatoms (in the NPSi-C treatment) and dinoflagellates (in the NP-C treatment) (Pearman et al., 2016). *Prochlorococcus* was a less abundant or minor component of the initial community, as reported for surface waters in other oligotrophic coastal areas (Veldhuis and Kraay, 1993; Babiæ et al., 2017; Pearman et al., 2017), and did not respond to nutrient additions as expected by its higher preference for ammonium instead of nitrate (Berube et al., 2015; Biller et al., 2015). On the other hand, the lack of nutrient addition in the control treatment precluded the development of phytoplankton and of *Synechococcus*.

The data derived from metagenomic analyses allowed a deep, unbiased characterization of the natural *Synechococcus* population to a clade level. *Synechococcus* clades I-IX had been previously observed in the Gulf of Aqaba, in northern Red Sea (Fuller et al., 2003, 2005; Lindell et al., 2005; Mühling et al., 2005; Penno et al., 2006; Post et al., 2011). In the present work, using more recent and complete *Synechococcus* clade databases, we could also identify the presence of clades XV, UC-A, WPC1, CRD1, and cluster 5.2.

Our results showed a prevalence of reads associated with clade II at the beginning of the experiment and during the blooms. Clade II is found in most (sub)tropical marine waters encompassing a broad range of nutrient concentrations (Post et al., 2011). This result also agreed with previous studies in the Red Sea (Fuller et al., 2003, 2005; Farrant et al., 2016), where this clade clearly prevailed in the *Synechococcus* community. Clades II and III (the second most abundant) have been reported to be prevalent and dominant in warm, oligotrophic, open-ocean habitats in tropical and subtropical oceans (Sohm et al., 2016), consistent with their dominant role in the Red Sea. Clade II also dominated the community forming a bloom in response to nutrient additions, whether delivered as a pulse of continuous additions. This can be observed here in the *Synechococcus* communities present in the four nutrient addition treatments on day 5, when the blooms were collapsing, and clade II remained as the dominant group (Tai and Palenik, 2009; Post et al., 2011). Other clades present at the beginning of the experiment also proliferated during the blooms, preserving similar proportions in the community. However, our results reflect a general decrease in the abundance of reads assigned to the different *Synechococcus* clades, following *Synechococcus* cell abundances decrease after the initial blooms in the nutrient addition treatments. Overall, the most conspicuous change is the decrease in the most abundant clade II and in its dominance in the population, while the proportion of other less abundant clades increased (e.g., UC-A), likely taking advantage as the resources not exploited by clade II became available. Clade II is the dominant *Synechococcus* clade in the Red Sea and the decline observed at the end of the experimental time reflect the general decrease of *Synechococcus* and competition with diatoms and other phytoplankton groups that proliferated in the mesocosms after the first week (Pearman et al., 2016). Besides this, low *Synechococcus* abundances toward the end of the experiment hampered a proper clade identification and were reflected in a higher proportion of unidentified clades, significantly in the continuous addition treatments.

In the control treatment, the predominating *Synechococcus* clades shifted from II to III, with clade III dominating the small bloom observed on the second week. Post et al. (2011) observed during a temporal study on the Gulf of Aqaba that while *Synechococcus* was dominated by clade II during the spring bloom, clade III contributed significantly to diversity during summer stratification (Mühling et al., 2005; Post et al., 2011). In fact, clade III became more prevalent in the Red Sea and in other oligotrophic areas during periods of severe nutrient depletion, occupying preferably stable, low-nutrient environments (Penno et al., 2006; Zwirglmaier et al., 2008; Post et al., 2011; Farrant et al., 2016). At the end of the experiment, nutrients were below detection limits in the control treatment, and the growth of clade III supports its preference for strong oligotrophic conditions. The proportion of clade III also showed a significant increase in NP-I, as nutrients became exhausted along the incubation, although in this case clade II remained dominant. In the *Synechococcus* populations present in nutrient addition treatments, increased cell abundances during the blooms allowed the quantification of the low abundant clade CRD1 in the initial addition treatments on day 5, but not in the continuous addition treatments. This difference contributed to the two slightly different outcomes for evenness between initial and continuous addition treatments observed on day 5, with decreased E_*H*_ values in NP-I and NPSi-I treatments.

At the end of the experiment, when blooms were finished for all the nutrient treatments, there was a slight reduction in clade richness. This was related in general to the decrease of clades XV, CRD1 and VII, the least abundant groups (<1% reads) that were not always detected along the experiment. Clade CRD1 appeared in the initial addition treatments, but not in the continuous addition treatments. In the non-blooming control treatment, the number of clades did not change. However, as clade III abundance increased, clade evenness also increased, and once clade III became predominant instead of clade II, evenness decreased again. In the continuous addition and NPSi-I treatments, evenness increased toward the end of the experiment as clade II dominance decreased. The result for NP-I differed as evenness decreased after the bloom but increased toward the onset of the experiment as the population of clade II recovered as observed at the beginning of the incubations. The reason for these changes could be associated to the cyanophage dynamics that were larger in this specific treatment.

Metagenomic data is superior to amplicon data (e.g., 16S or similar) in that it is PCR-free and, therefore, is free of the biases derived from differential amplification of sequences from different clades and viruses. Concurrently, metagenomic analyses provide information on the abundance for cyanobacteria and their associated phage community, as number of reads assigned to the organisms of interest. A high presence of viral DNA can be found in cell fraction metagenome samples (>0.2 μm), containing key viral information, which may be missing in viriome studies (e.g., Ghai et al., 2010; López-Pérez et al., 2017). The viral genes detected in the cellular fraction correspond to viruses on an intracellular stage (including replication intermediates generated during the lytic cycle, prophages or viruses on a lysogenic stage), big-sized viruses, viruses attached or adsorbed to particles, and free viruses that are retained as the filter saturates (De Long et al., 2006; Rodriguez-Valera et al., 2014; López-Pérez et al., 2017). This technique allowed us to get detailed information to differentiate and identify the *Synechococcus* cyanophages from other phages present in our samples, as bacteria are the most abundant organisms in the oceans (Cochlan et al., 1993; Fuhrman, 1999; Weinbauer, 2004), from which the ubiquitous *Synechococcus* is only a fraction (Li, 1998; Li and Harrison, 2001).

Our metagenomic approach revealed a massive cyanophage infection developing a few days after the onset of the *Synechococcus* bloom. Phage infection is a significant source of mortality of marine microorganisms (Proctor and Fuhrman, 1990; Fuhrman and Suttle, 1993; Suttle and Chan, 1994), exerting a major influence in their community structure, diversity and dynamics (Suttle et al., 1990; Bratbak et al., 1993; Brussaard et al., 1996). It has been estimated that about 15% of marine cyanobacteria are infected by phages at any given time (Proctor and Fuhrman, 1990), and that approximately a 2–3% of *Synechococcus* primary production is daily lost to viral lysis (Suttle, 1994). Viral reproduction requires host cell infection; infectivity increases with host abundance because infection is a direct function of the encounter rate between a pathogen and its host (Danovaro et al., 2011). Viral abundances increase in response to the increasing host abundances during algal blooms (Bratbak et al., 1990; Suttle and Chan, 1994; Weinbauer and Suttle, 1996), and has been reported to be a major factor responsible for bloom terminations. For example, Nagasaki et al. (1994) described the role of viruses in the rapid termination of a red-tide algal bloom in the Inland Sea of Japan. Also, several studies have related the cell mortality caused by viruses with the termination of blooms of the haptophyte *Emiliana huxleyi* in the North Sea (Bratbak et al., 1993; Brussaard et al., 1996; Jacquet et al., 2002). Grazing by protists could be also responsible for the decline of *Synechococcus* blooms, as both viral infection and grazing exert control over picoplankton populations (Baudoux et al., 2008; Boras et al., 2009). However, the evolution of protist abundances during our experiments did not show any negative relationship to *Synechococcus* abundances (Pearman et al., 2016), increasing their abundance toward the end of the first week of incubation, once the *Synechococcus* blooms had already collapsed. Therefore, our results are consistent with published reports indicating that nutrient amendments stimulate lytic phage production as a result from the enhanced growth of host cells (Williamson and Paul, 2004). The majority of cyanophages that have been isolated are lytic (Suttle, 2005). However, in natural populations of *Synechococcus*, the lysogenic phase prevails at times of low host availability, resource limitation or adverse environmental conditions in order to ensure viral survival (McDaniel et al., 2002; Ortmann et al., 2002), as has been observed for different bacterial communities (Weinbauer et al., 2003; Payet and Suttle, 2013). During lysogeny, the genetic material if the phage is integrated into the host genome as a prophage and subsequently transmitted vertically during cell division in a non-infectious form. Later, the lytic pathway can be induced either spontaneously or by physical, chemical or biological factors, such as increased growth of the host (Paul, 2008; Payet and Suttle, 2013). As observed here, when resource supplies allow the host concentration to proliferate, concentrations of cyanophages increase dramatically (Suttle and Chan, 1994; Weinbauer and Suttle, 1996), thereby acting as key drivers in terminating algal blooms (Wilson and Mann, 1997; McDaniel et al., 2002; Mühling et al., 2005).

Viruses can exert a significant selection pressure on the host community (e.g., Suttle and Chan, 1993, 1994; Ahlgren et al., 2019). *Synechococcus* phages show varied host specificities, being able to attack one or several host strains (Suttle and Chan, 1993), with a strong effect on the community microdiversity of the host (Ahlgren et al., 2019). The viral community also changes, as observed in Alarcón-Schumacher et al. (2019), who described a shift in the predominant viral family from Myoviridae, that infects bacteria and archaea, to Picodnaviridae, infecting eukaryotes, during a diatom-dominated bloom. In our study, the most abundant *Synechococcus* phages were T4-like phages, belonging to the widely distributed Myoviridae family (Mann, 2003; Ma et al., 2014). T4-like myoviruses can encode their own tRNAs, a strategy that may allow them to expand their potential host range (Limor-Waisberg et al., 2011), supporting the hypothesis that in oligotrophic oceanic waters, phages with broad host ranges would have a greater chance of successful host encounter (Wommack and Colwell, 2000). This way, as the host-phage interactions in the experimental blooms reported here are unlikely to be strain-specific but mainly density-dependent, the most successful host lineage will be preferentially attacked by phages (Thingstad et al., 2008). Consistently, during our study the viral attack seems to have affected the most abundant clade (clade II), whose proportion in the *Synechococcus* population showed a decreasing trend after the bloom had started to collapse.

Phage-host interactions influence host population diversity, in terms of the number of taxa present (richness) or the homogeneity of their frequencies (evenness) (Mühling et al., 2005; Rodriguez-Valera et al., 2009). The Shannon’s equitability index decreased in all treatments as the number of *Synechococcus* reads increased, relating homogeneity to the development of blooms and the changes in the dominance of clade II. The sharper increase in evenness between days 5 and 8 for the initial addition treatments could be related to the phage-induced mortality of clade II, which grew again at the end of the experiment once virus numbers decreased, resulting in a decrease in evenness. This contrasts with the control treatment, where the absence of nutrient amendments prevented the development of a *Synechococcus* bloom, and the less pronounced viral control allowed clade III (better adapted to low nutrient conditions) to thrive, replacing clade II.

As active members of marine planktonic communities, viruses influence community composition introducing organic nutrients into the system that can be recycled by bacteria (Lara et al., 2017), and also by removing the fast-growing, blooming hosts (Bratbak et al., 1990; Fuhrman, 1999). During our experiment, different picoeukaryotes and nanoeukaryotes increased their abundance to dominate the community following *Synechococcus* bloom collapse (Pearman et al., 2016), as observed in previous mesocosm experiments (Duarte et al., 2000; Castberg et al., 2001). Hence, our results suggest viral control of *Synechococcus* abundances probably had indirect consequences on community structure and dynamics, providing opportunities and recycled resources for other species to grow (Castberg et al., 2001).

In summary, our metagenomic analysis showed that intense nutrient inputs triggered *Synechococcus* blooms, without conspicuous changes in clade composition. These blooms appear to have been terminated by subsequent cyanophage outbursts. The observed decrease in *Synechococcus* clade II after the blooms had collapsed increased the evenness within the *Synechococcus* population, more strongly in the initial addition treatment where the preferential viral attack on the most abundant clade may have accentuated the change in the host evenness. In contrast, during the control treatment, the low nutrient availability prevented the development of a *Synechococcus* bloom and of the consequent phage outburst, with a substantial change in *Synechococcus* clade composition where clade III outcompeted clade II.

These results reflect the dynamic composition of *Synechococcus* communities and highlight the importance of nutrient-induced blooms and viral infection in maintaining *Synechococcus* abundance and diversity, with broad implications for the dynamics of plankton communities in warm, oligotrophic waters.

## Data Availability Statement

The metagenomic data analyzed here has been submitted to the ENA archive under the accession number PRJNA395437, and is also available for public access through DMAP (http://www.cbrc.kaust.edu.sa/dmap), in project 55, under the name “Red Sea MESOCOSM samples (Gene Abundance).”

## Author Contributions

AC-C, CD, SA, and XI designed the study. XI and JP executed the experiment and obtained the data. AC-C, RD-R, CD, and SA wrote the manuscript. All authors revised and approved the manuscript and contributed to data analysis.

## Conflict of Interest

JP was employed by Cawthron Institute.

The remaining authors declare that the research was conducted in the absence of any commercial or financial relationships that could be construed as a potential conflict of interest.

## Footnotes

1 http://www.cbrc.kaust.edu.sa/dmap
